# Proverbs and Aphorisms in Neurorehabilitation: A Literature Review

**DOI:** 10.3390/ijerph18179240

**Published:** 2021-09-01

**Authors:** Roberto Cano-de-la-Cuerda

**Affiliations:** Motion Analysis, Ergonomics, Biomechanics and Motor Control Laboratory (LAMBECOM), Department of Physical Therapy, Occupational Therapy, Rehabilitation and Physical Medicine, Universidad Rey Juan Carlos, Alcorcón, 289122 Madrid, Spain; roberto.cano@urjc.es; Tel.: +34-914-888-674

**Keywords:** neurorehabilitation, neurosciences, proverbs, aphorisms, brain, neuroplasticity, neurological disorders

## Abstract

Introduction: Brain plasticity is not limited to childhood or adolescence, as originally assumed, but continues into adulthood. Understanding this conceptual evolution about the nervous system, neuroscience and neurorehabilitation, researchers have left different proverbs and aphorisms derived of their investigations that are still used in university and postgraduate training. A proverb is defined as a phrase of popular origin traditionally repeated invariably, in which a moral thought, advice or teaching is expressed. On the other hand, an aphorism is understood as a brief and doctrinal phrase or sentence that is proposed as a rule in some science or art. The aim of this paper is to present a compilation of proverbs and aphorisms related to neuroscience and neurorehabilitation, classified chronologically, to illustrate the conceptual evolution about the brain and to improve our understanding about the management of neurological patients through the methods and techniques developed during the 19th, 20th and 21st centuries, as many therapies are based on them. Methods: A literature review was conducted based on the recommendations for Systematic Reviews guidelines for scoping reviews. A computerized search was conducted in the following electronic databases: CINAHL Medical Science, Medline through EBSCO, PubMed, Physiotherapy Evidence Database (PEDro) and Scopus, limiting the search to papers published until April 2021 in English and Spanish. Inverse searches were also carried out based on papers found in the databases. The following data were extracted: technique or approach; author; date of birth and death; proverbs and aphorisms; clinical interpretation. Results: Proverbs and aphorisms linked to authors such as Charles Edward Beevor (1854–1908), Heinrich Sebastian Frenkel (1860–1931), Rudolf Magnus (1873–1927), Nikolai Bernstein (1896–1966), Donald O. Hebb (1904–1985), Elwood Henneman (1915–1996), Wilder Graves Penfield (1891–1976), Humberto Augusto Maturana Romesín (1928), Edward Taub (1931), Janet Howard Carr (1933–2014), Roberta Barkworth Shepherd (1934), Brown & Hardman (1987), Jeffrey A. Kleim and Theresa A. Jones (2008) were compiled. Conclusion: Different authors have developed throughout history a series of proverbs and aphorisms related to neurosciences and neurorehabilitation that have helped to better our understanding of the nervous system and, therefore, in the management of the neurological patient through the methods and techniques developed throughout the 19th, 20th and 21st centuries.

## 1. Introduction

Rehabilitation is defined by the World Health Organization (WHO) as “an active process through which people disabled as a result of illness or injury, achieve full recovery, or if full recovery is not possible, develop their maximum physical, mental and social potential, and are integrated in the most appropriate environment” [1]. Therefore, the diagnosis, evaluation, prevention and treatment of disability is closely linked to the concept of rehabilitation. In this context, the current epidemiological situation related to neurological disorders shows that neurorehabilitation is key to minimizing alterations in structure and function, limitations in activity and restrictions in the participation of subjects with such conditions [2].

The plastic capacity of the human nervous system was not recognized until recently; thus, the scientific origins of neurological rehabilitation are relatively new [3,4,5]. The term neurorehabilitation is understood as a process aimed at reducing the deficiency, limitation of activity and restriction of participation suffered by people as a consequence of a neurological disease. Professionals involved in this field aim to reduce the degree of functional impairment of the patient. This is an educational and dynamic process based on the adaptation of the individual and their environment to their neurological deterioration [4].

Neurorehabilitation aims to improve the independence and quality of life of patients by reinforcing their skills and encouraging participation. The WHO defined it as “an active process through which people disabled due to injury or neurological disease achieve a complete recovery or if this is not possible, they can take advantage of their optimal physical, mental and social potential and integrate into the environment more appropriate” [3]. Among its main objectives are to reduce the impact of the disease on the person and their environment and to improve their quality of life related to health within the limitations imposed by the neurological deficit [4,5,6].

For many years, the central nervous system was considered a functionally immutable and anatomically static structure [7]. The dogma “no new neurons” used during that time also meant “no new connections”. Thus, once embryonic development was complete, the system was considered a finished and definitive entity, mutable only by injury or degeneration and irreparable by its very nature. Ramón y Cajal wrote “...the functional specialization of the brain imposes two great gaps on neurons: inability to proliferate and irreversibility of intraprotoplasmic differentiation. It is for this reason that, once development is complete, the sources of growth and regeneration of axons and dendrites dry up irrevocably. In adult brains the nerve pathways are something fixed, finished, unchanging. Everything can die, nothing can regenerate. It is up to the science of the future to change, if possible, this cruel decree” [8,9]. Although a static and invariable brain was classically conceived, we now know that this is not the case. Brain plasticity is not even limited to childhood or adolescence, as originally assumed, but continues into adulthood [10,11,12].

Understanding this conceptual evolution about the nervous system, neuroscience and neurorehabilitation, researchers have left different proverbs and aphorisms derived of their investigations that are still used in university and postgraduate training. A proverb is defined as a phrase of popular origin traditionally repeated invariably, in which a moral thought, advice or teaching is expressed [13]. On the other hand, an aphorism is understood as a brief and doctrinal phrase or sentence that is proposed as a rule in some science or art [14]. Therefore, the aim of this paper is to present a compilation of proverbs and aphorisms related to neuroscience and neurorehabilitation, classified chronologically, to illustrate the conceptual evolution about the brain and to improve our understanding about the management of neurological patients through the methods and techniques developed during the 19th, 20th and 21st centuries, as many therapies are based on them (Table 1) [15].

## 2. Materials and Methods

### Study Design

The methodology for this literature review was based on the recommendations for Systematic Reviews guidelines for scoping reviews. Three authors performed article screening, data extraction and determined the presence of proverbs and aphorisms linked to neurosciences and neurorehabilitation techniques or approaches. Discrepancies were resolved through discussion, and a fourth author was consulted if needed.

In this literature review, the presence of proverbs and aphorisms described were searched and linked to the main rehabilitation techniques or approaches described in Table 1. As a first attempt, a list of techniques was compiled based on a previous work by Florez [15]. Later, the list was revised, and additional approaches were identified and included. A computerized search was conducted in the following electronic databases: CINAHL Medical Science, Medline through EBSCO, PubMed, Physiotherapy Evidence Database (PEDro) and Scopus, limiting the search to papers until April 2021 in English and Spanish. Inverse searches were also carried out based on papers found in the databases.

For each proverb and aphorism identified and described by an author, historical background and its contribution based on human or animal studies were analyzed. We focused on reviews, perspectives and debates around bases and foundations of rehabilitation methods, techniques and concepts for people with neurological disorders. Study protocols or clinical trials, prevention methods, pharmaceutical or other medical interventions were excluded.

The following data were extracted: technique or approach; author; date of birth and death; proverbs and aphorisms; clinical interpretation.

## 3. Results

A total of 100 studies were initially identified. After excluding duplicates, 50 were sieved by title and abstract, and 14 studies were ruled out since they did not satisfy the eligibility criteria. Finally, 36 papers were included in this literature review [16,17,18,19,20,21,22,23,24,25,26,27,28,29,30,31,32,33,34,35,36,37,38,39,40,41,42,43,44,45,46,47,48,49,50,51].

Proverbs and aphorisms linked to authors such as Charles Edward Beevor (1854–1908), Heinrich Sebastian Frenkel (1860–1931), Rudolf Magnus (1873–1927), Nikolai Bernstein (1896–1966), Donald O. Hebb (1904–1985), Elwood Henneman (1915–1996), Wilder Graves Penfield (1891–1976), Humberto Augusto Maturana Romesín (1928), Edward Taub (1931), Janet Howard Carr (1933–2014), Roberta Barkworth Shepherd (1934), Brown & Hardman (1987), Jeffrey A. Kleim and Theresa A. Jones (2008) were compiled.

## 4. Discussion

### 4.1. Charles Edward Beevor (1854–1908)

The English anatomist and neurologist developed his axiom, known as the Beevor Axiom in 1903, based on experiments showing that, when an area of the cortex was stimulated, the body responded with a global movement, not just a muscle. His Axiom stated that “the brain does not know the action of the isolated muscle but the movement” [16]. For this reason, the coordination between the different muscular movements and actions with a functional objective in rehabilitation is preferable than the training of isolated muscles. The brain is organized into movement patterns to generate sequences, such as walking, dressing, professional and sporting gestures or putting food in the mouth, since less brain activity is needed to carry it out. This is compatible with the Beevor Axiom, as the brain is able to evoke and reproduce movements more easily than it takes to learn them. “Only coordinated movements are represented in the excitable cortex” This was collected by Voss in 1991 as the basis of proprioceptive neuromuscular facilitation techniques, through retraining of diagonal and spiral movement patterns, as the basis of all functional movement of the head, trunk and extremities [17], which are used in Neurorehabilitation Units.

### 4.2. Heinrich Sebastian Frenkel (1860–1931)

Frenkel is considered a pioneer of neurorehabilitation [18,19,20]. He was the first to introduce the concept of exercise to restore dexterity and to improve coordination and ambulation. Frenkel stated that “the treatment is effective not by muscular work, but by the repetition of an active voluntary movement” [18]. This constitutes one of the foundations of functionality training in the patient with neurological pathology, which is the basis of neurorehabilitation today. His original experience with a patient with tabes dorsalis, described in his manual “Treatment of Tabetic Ataxia by Means of Systematic Exercise” [21], led him to consider that both the subject’s motivation and repetition (training) were key elements in the functional improvement of the patient. Based on this, he devised a method based on repetition (elaboration of exercises from the simplest to the most complex), feedback (visual and auditory), function (retraining functional tasks as the basis of treatment) and motivation (active participation) by patients, as key elements in neurorehabilitation, which have broad support in current scientific evidence [18]. In fact, he bequeathed the following phrase “the goal of Übungstherapie (neurological gymnastics) is not to strengthen muscles, but to build on the most important characteristic of the nerve substance, its ability to improve through exercise” [19]. As has been indicated in previous publications [18], such a statement implied a primal vision of what most of the physical treatments in neurorehabilitation have subsequently assumed, based on advances in the sciences of motor control and learning. It is known in the context of the neurorehabilitation of skills and abilities lost that the active practice of activities are relevant, through the establishment of tasks that involve repetition.

### 4.3. Rudolf Magnus (1873–1927)

Magnus was a German pharmacologist and physiologist. The way in which the nervous system organizes us, through postural adjustments and the generation of movement, was defined by his Rule of the Deviation of Magnus, “at all times, and before any postural change, or movement, the Nervous System responds by organizing a postural or motor adjustment response according to the alignment of the musculoskeletal components” [22]. That is, at all times, the nervous system records the situation of all musculoskeletal components within our body scheme and in relation to the outside world (spatial scheme). Therefore, “the Central Nervous System knows faithfully and at all times the state of the musculature”. The motor gesture does not occur exclusively in relation to the knowledge and storage of motor patterns but also through interaction with the alignment of the different segments of our body. Thus, each joint, with all the musculoskeletal components that make it up, are responsible not only for producing movement but also for sending a large amount of information through the receptors [23,24]. As mentioned in the Magnus Rule, muscle tone must be able to be variable to adapt to the alignment needs of the musculoskeletal system to produce adequate responses [25], transmitting a change to the central nervous system as soon as it is perceived, thus causing a change in muscle tone. This aphorism was integrated into the Bobath Concept, popularly applied in neurorehabilitation.

### 4.4. Nikolai Bernstein (1896–1966)

Nikolai Alexandroviþ Bernštein was born in Russia in 1896 and developed his professional activity in the former Soviet Union. He dedicated a good part of his professional life to the study of the physiological mechanisms of human movement. According to this author, the neural control of movement could not be understood without understanding the characteristics of the systems that move. He affirmed that “movements are neither centrally nor peripherally directed, but rather emerge from the interaction of many systems” [26]. His systems theory considers the whole body as a mechanical system subject to external forces, such as gravity, and internal forces, such as muscular actions. During the course of any action, the forces acting on the body change, which may cause different movements, to achieve the same objective due to the interaction between external and internal forces, as well as variations in the initial conditions or that the same movement could be caused by different commands. Therefore, since the body has high degrees of freedom that need to be controlled, the higher levels of the nervous system would activate the lower ones, which would activate the synergies or groups of muscles forced to act together as a unit [27]. This author enunciated the phrase “practice is a particular way of repeating without repeating”, that is, practice, to be acquired and perfected, must present a control of the parameters that could be modified, presenting itself as a challenge for the patient to be extrapolated to different environments and situations. The practice of movements should not have the purpose of accumulating but rather to favor that the subject is able to respond adequately to the changes that may arise in the course of carrying out the tasks [26,27,28]. The Bobath Concept, modern techniques and some technologic approaches have integrated this aphorism into their theoretical foundation.

### 4.5. Donald O. Hebb (1904–1985)

The WHO defined in 1982 the term neuroplasticity as “the capacity of the cells of the nervous system to regenerate anatomically and functionally, after being subjected to environmental or developmental pathological influences, including trauma and disease. This allows an adaptive (or maladaptive) response to functional demand” [26]. However, to arrive at this current conceptualization of the term, many scientists and clinicians worked to verify the dynamic and plastic capacity of the nervous system. Donald O. Hebb is considered the father of biopsychology and formulated his Hebbian Theory, consisting of the ability of our brain to arm, strengthen, disarm and weaken neural networks, through the aphorism “the cells that fire together, they will remain connected” [29,30]. “The idea is old, two cells or cell systems that are continuously active at the same time, will tend to become associated, so that the activity of one will facilitate that of the other” However, “if a Hebbian network is not used, it will gradually lose its component cells, until it disappears”. Therefore, “synapses are removable by experience” [29,30].

### 4.6. Elwood Henneman (1915–1996)

The spinal motor neurons that innervate the muscles are heterogeneous since they present variations in the diameter of their cell bodies, axons, dendritic trees, as well as in the contractile properties of the associated muscle fibers. Therefore, the question was how and which motor neurons are selected by the central nervous system to produce the different types of contractions and movements. Elwood Henneman tried to answer this through Henneman’s Size Principle (1957), which indicated that for any net contribution of excitation to a group of motor neurons, “the motor neurons are recruited in an orderly manner, always in small to large” [31]. It is important to indicate that the recruitment ordered by size was originally defined for movement in conditions without pathology and for isometric contractions (not being extrapolated in eccentric contractions or in rapid or ballistic movements). Therefore, in conditions of a physiological “noisy” system, as in the processes of aging, reinnervation, pain or fatigue when the precision decreases, or in subjects with pathology, other processes can alter the recruitment ordered by size. However, under laboratory conditions, with simple muscles and a limited set of contractions, recruitment is orderly in terms of force [32].

In biomechanically complex musculoskeletal systems, accurately measuring the size of a motor unit is difficult. Therefore, the question remains whether the size principle, defined under laboratory conditions for orderly recruitment, is generalizable to daily functional movements [33]. This principle of order of muscle contraction has been one of the foundations of therapeutic approaches in neurorehabilitation, such as the Bobath Concept, explaining the phenomena of stability prior to the movement of other body segments. However, recent reviews seem to point out that the Henneman Principle would not be fulfilled if we take into account the same muscle, as suggested by said researcher. In addition, Henneman experimented with isolated muscle preparations, which is not necessarily extrapolated to a complex physiological system, in which selective recruitment patterns can be generated according to the type of movement (speed, direction, among others). Likewise, this principle would not be verified in the primary respiratory muscles during voluntary and involuntary breathing or under electrostimulation conditions [34], very common in Neurorehabilitation Units.

### 4.7. Wilder Graves Penfield (1891–1976)

The knowledge of the cortical representation of the movement comes fundamentally from the works of Penfield in the first half of the 20th century [35]. He identified a unique and somatotopic representation of the different parts of the body and postulated that the organization of movement followed a sequential order. Thus, in 1937 Penfield and Boldrey presented a cortical map represented as a doll called “homunculus” (“little man”) that represented the cortical areas corresponding to the different parts of the body. In 1950, Penfield and Rasmussen specified the organization of that first homunculus, obtaining the first somatotopic map of the motor and sensory cortex separately, with a proportionally greater representation of the parts that perform finer and more precise movements for the motor cortex, or directly proportional to the number of specialized sensory receptors in each peripheral zone of the organism for the sensory [26].

As Sallés et al. [36] indicate, Penfield’s work was questioned, and even Penfield himself warned of the possible inaccuracy of his maps. The first articles that did so appeared in the 1980s by Strick and Preston and exposed the existence of two representations at the cortical level of the hand in the brain of a primate. “There is a multiple cortical representation depending on the use made of the movement”. This statement was considered disruptive because up to that moment there was only talk of a representation of the hand in the brain of the human being.

Subsequently, Mezernich and Kaas in 1982, after carrying out their studies and research, described twelve representations of the hand, which were presented according to use. Therefore, there would be representations dependent on functionality and experience, developed during the activity carried out with the hand. According to Kaas “the representations would be related to the use. The same movement, for different tasks, would activate different brain areas” [37]. This was extrapolated by Gould and Koll in 1986 [38], to a multiple cortical representation, not only of the hand but of the different parts of the body such as the foot, trunk or lower limb. In summary, and quoting Asanuma in 1972, “in motor behavior the dissociation of motor and sensory aspects is artificial, if not impossible”. Cognitive approaches, such as Perfetti technique, uses these aphorisms to justify this therapy.

### 4.8. Humberto Augusto Maturana Romesín (1928)

The Neurocognitive Theory of Rehabilitation was born in the seventies within a working group led by Professor Perfetti, under the conviction that the recovery process after an injury is directly influenced by the activation of the subject’s cognitive processes [5]. The theory evolves in parallel with the development of cognitive neuroscience, where thanks to progress in neuroimaging techniques it has been possible to delve into the study of the functioning of the central nervous system on the processes of perception, cortical organization of voluntary movement, attention, problem solving, and evocation of motor images, among others.

The Neurocognitive Theory maintains that the entity and the qualitative level of the patient’s recovery, whether spontaneous or guided by the rehabilitative intervention, is determined by the cognitive processes activated by the patient and by the modality in which they come activated. The cognitive processes that are considered necessary to achieve a qualitative recovery in pathological conditions are mainly attention, memory, perception, sight, representation and language [5]. The Chilean biologist Humberto Maturana even relates them to the idiosyncrasy of living beings, since they are characterized by their ability to learn through them. In this context, Maturana stated in 1990 that “any biological alteration of our Nervous System modifies our cognitive capacity and vice versa”. Neurocognitive therapeutic approaches, such as the Perfetti method, emphasize the influence of these cognitive processes on the quality of recovery, underlining their link in the organization of the nervous system [37]. Perfetti’s maxim will be that the recovery process will be understood as a learning process that takes place in pathological conditions, where movement considered a means of knowing the environment that surrounds the individual and the body is interpreted as a receiving surface of information. Carlo Perfetti bequeaths the phrase that is undoubtedly picked up by the therapists who apply his method: “the patient does not learn to move by moving, but by perceiving” [37].

In addition, an important factor to take into account in relation to the exercises proposed by Perfetti is provided by Mountcastle. This author indicates “attention is important in the organization of movement activating diverse cortical areas” [37]. This author verified how the same movement, performed with different attention in each case, modified and activated different cortical areas. An example is taking a bottle with the intention of checking whether it is full or empty, or to see if the liquid inside is cold or hot; the stimulated cortical area is actually modified, even making the same movement, since the intention and attention are different in one case than in the other. Therefore, “the neural network is covered in a different way according to the type of attention proposed, according to the information preferably used, according to the purpose and use made of the movement”, as is reflected within the Perfetti technique [5,37].

### 4.9. Edward Taub (1931)

Research on behavioral interventions in monkeys led to a new and effective group of techniques for motor rehabilitation after stroke, in cases of cerebral palsy, head trauma and other neurological injuries in humans. These approaches, which involve repetitive training of the paretic limb in task-oriented activities, have been shown to be effective in those patients who meet certain characteristics. Among these interventions is Constraint-Induced Movement Therapy [5]. This term is used to denote a set of treatment modalities whose common characteristic is to discourage the use of the unaffected or less affected upper limb, combining it with intensive training of the paretic limb.

Constraint-Induced Movement Therapy is capable of producing changes in brain organization and function. The use of this brain plasticity through the application of behavioral techniques promises the development of new treatments in the field of rehabilitation. The Constraint-Induced Movement Therapy is, essentially, a behavioral intervention, whose procedures are relatively simple and effective, since they produce both an increase in the use of the affected limb in daily activities, as well as the persistence of the effect of the treatment in the short and medium term [5]. It is based on the research carried out by Dr. Taub in the late 1970s and early 1980s, who enunciated the learned non-use theory. This theory is based on experiments carried out with monkeys in which a limb was deafferented by a dorsal rhizotomy. As a result of the loss of sensory feedback, the monkeys did not use that limb again, unless its use was forced by restricting the movement of the healthy limb. If the restriction of the healthy limb was maintained for one to two weeks, there was a permanent change in the use capacity of the disaffected limb [5]. Learned disuse develops during the initial phase after central nervous system injury. According to Taub, the injured animal (or the stroke patient) “learns to avoid the affected limb as a result of a learning process” in which attempts to use said limb are negatively reinforced by the consequences of the attempts (for example, falls or failures to achieve a goal). Although the ability to handle the affected limb gradually recovers over a period of weeks or months, the learned disuse behavior remains, and its utilization is much less than it potentially could be. In conclusion, stroke patients learn to disuse and must therefore overcome the learned disuse theory [39,40,41,42].

### 4.10. Janet Howard Carr (1933–2014) and Roberta Barkworth Shepherd (1934)

In 1984, two Australian physiotherapists, Carr and Shepherd, drawing on studies of movement science, neurophysiology and learning theories, proposed a new approach to the re-education of stroke patients. According to Carr and Shepherd, the goal of rehabilitative treatment should be re-learning oriented to specific tasks, forcing the use of the paretic side, since its principle was simple and intuitive: “one learns what one practices” [43,44].

Activity-oriented methods (Table 1) rely on the recognition that the goal of motor control is mastery of movement to perform a particular action, not to perform movements for the sake of moving. Thus, movement control would be organized around goal-directed functional behaviors. It remains to be agreed which are the fundamental activities of the nervous system and the essential elements that are controlled in an action [5].

Other modern techniques such as body-weight-supported treadmill training and constraint-induced movement therapy, or technologic approaches such as robotics, virtual reality, and video-games, are based in this clear aphorism proposed by Carr and Shepherd.

### 4.11. Brown & Hardman (1987)

Neurorehabilitation treatment must be early, supported by the plasticity of the nervous system, and based on the principle that the form and function of the central nervous system form a unit. The anatomy of the form determines the function. The function is a demand on the central nervous system and represents a stimulus for the creation of new neural connections, therefore, to modify the anatomical shape. The modified form, in turn, leads to a modified function, and so on [45].

According to Brown and Hardman: “The function changes if the form changes, the form changes if the function changes”. This plastic capacity of each cell of the organism to organize and reorganize itself again in each phase of its development allows the germination of dendrites and axons, thus forming new synapses and thus making new connections with other cells [45,46]. This aphorism is picked up by the Bobath Concept in their Neurodevelopment Treatment (NDT).

### 4.12. Jeffrey A. Kleim and Theresa A. Jones (2008)

Kleim and Jones, researchers from the University of Florida and the University of Texas, published in 2008 their decalogue of aphorisms related to neuroplasticity that are currently followed by all neurorehabilitation professionals [48]: “Use it or you will lose it; Use it and improve it; Specificity in training matters; Repetition matters; Intensity matters; The timing matters; Relevance (motivation and attention) matters; Age matters; Transfer matters; Interference matters”.

Different investigations show that for there to be objective learning and re-learning, that is, for changes in neuronal architecture to occur, functional, repeated, rewarded and carried out activities must be carried out over time, for which neurorehabilitation programs these elements should be considered in therapy [49]. For this reason, the decalogue of aphorisms proposed by Kleim and Jones is integrated into most of the clinical practice guidelines used in neurorehabilitation units for patients with neurological pathology [50,51] and almost all neurorehabilitation techniques based on neuroplasticity and motor learning.

## 5. Conclusions

Despite the fact that the term neurorehabilitation is relatively recent, there has been a conceptual evolution fundamentally linked to a greater knowledge about the plastic capacity of the nervous system. Different authors have developed throughout history a series of proverbs and aphorisms related to neurosciences and neurorehabilitation that have helped to better understand the nervous system and, therefore, in the management of the neurological patient through the methods and techniques developed throughout the 19th, 20th and 21st centuries.

## Figures and Tables

**Table 1:** Historical and conceptual classification of techniques in neurorehabilitation.

	Description	Aim	Approaches and Techniques
<1940	Compensation techniques	Stimulate unaffected side	
1940–1960	Facilitation techniques	Improve quality of movement on the affected side	Proprioceptive neuromuscular facilitation 1940 Bobath concept 1948 Brunnstrom 1950 Rood Method 1954
>1970	Neurocognitive techniques	Introduction of cognitive functions in the rehabilitation process	Perfetti Method 1970
>1970 to 1980	Modern techniques	Motor learning	Task-oriented motor learning 1984 Body-weight–supported treadmill training 1987 Constraint-induced movement therapy 1970–1980 Muscle strengthening programs and physical reconditioning 1970–1980
>1970	Technologic approaches	Sensory and motor learning, functional recovery and compensation approaches	Robotics Virtual reality Video games Functional electrical stimulation Brain-Computer interface Transcranial magnetic stimulation Transcranial direct current stimulation Mobile applications Telerehabilitation

Modified from reference [15].

## Data Availability

Data is contained within the article.
